# Biochemical analysis of cross‐feeding behaviour between two common gut commensals when cultivated on plant‐derived arabinogalactan

**DOI:** 10.1111/1751-7915.13577

**Published:** 2020-05-09

**Authors:** Jose Munoz, Kieran James, Francesca Bottacini, Douwe Van Sinderen

**Affiliations:** ^1^ Microbial Enzymology Group Department of Applied Sciences Northumbria University Newcastle Upon Tyne NE1 8ST UK; ^2^ School of Microbiology & APC Microbiome Ireland University College Cork Ireland University College Cork Cork Ireland

## Abstract

In this paper, we reveal and characterize cross‐feeding behaviour between the common gut commensal *Bacteroides cellulosilyticus* (Baccell) and certain bifidobacterial strains, including *Bifidobacterium breve* UCC2003, when grown on a medium containing Larch Wood Arabinogalactan (LW‐AG). We furthermore show that cross‐feeding is dependent on the release of β‐1,3‐galacto‐di/trisaccharides (β‐1,3‐GOS), and identified that the *bga* gene cluster of *B. breve* UCC2003 allows β‐1,3‐GOS metabolism. The product of *bgaB* is presumed to be responsible for the import of β‐1,3‐GOS, while the *bgaA* gene product, a glycoside hydrolase family 2 member, was shown to hydrolyse both β‐1,3‐galactobiose and β‐1,3‐galactotriose into galactose monomers. This study advances our understanding of strain‐specific syntrophic interactions between two glycan degraders in the human gut in the presence of AG‐type dietary polysaccharides.

## Graphical abstract

In this paper, we reveal and characterise cross‐feeding behaviour between the common gut commensal *Bacteroides cellulosilyticus* and certain bifidobacterial strains, including *Bifidobacterium breve* UCC2003, when grown on a medium containing Larch Wood Arabinogalactan.

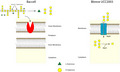

## Introduction

Arabinogalactans (AGs) represent plant cell wall‐associated proteoglycans which are widely distributed in the plant kingdom (Fujita *et al.*, 2019). The carbohydrate component of AGs is composed of a β‐1,3‐galactan backbone with β‐1,6‐galactan side chains which typically contain further decorations, together representing over 90% of the total AG mass. These decorations, which include, among others, l‐arabinose, l‐rhamnose, l‐fucose and d‐(methyl)‐glucuronic acid, are highly variable in length, linkage and sugar moiety (Ponder and Richards, 1997; Fujita *et al.*, 2019). The glycan component of such AGs will resist digestion by human enzymes to become available to the gut microbiota as fermentable substrates. Because of their complexity, it is likely that only a small number of gut bacteria are able to access the glycan component of AG either directly or indirectly by means of syntrophic interactions. If (some of) these AG‐metabolizing microbiota members convey health benefits to their host, such carbohydrates may be considered prebiotic (Harris *et al.*, 2019). Some AGs, exemplified by the glycan isolated from Larch Wood, are considered simpler in their chemical structure, whereas others, such as Gum Arabic from the Acacia Senegal tree, are considered more complex (Cartmell *et al.*, 2018). The latter AG (referred to as GA‐AG) is used extensively in the food industry as emulsifier (Muñoz‐Muñoz *et al.*, 2017), while the former has been commercialized as a fibre with purported prebiotic activity (Fujita *et al.*, 2014). The common use of AGs as food additives, and their ubiquitous presence in plant species, explains why these glycans constitute common components of the human diet.

The human gut microbiota (HGM) is an important contributor to human health where it supports and enhances the physiology of its host (Ndeh *et al.*, 2020; Glowacki *et al.*, 2020). The major nutrients available to this diverse microbial assembly are complex host‐ and diet‐derived glycans (Wang *et al.*, 2019; Ndeh *et al.*, 2020; Glowacki *et al.*, 2020). Consistent with the importance of glycan metabolism, genomic data revealed that members of the HGM encode a very large number of carbohydrate‐active enzymes (CAZymes), primarily glycoside hydrolases (GHs) and polysaccharide lyases (PLs). These enzymes are grouped into sequence‐related families and deposited in the CAZy database (Terrapon *et al.*, 2018). The structural fold, catalytic apparatus and mechanism are conserved within families, while substrate specificity may vary among members of a given family, exemplified by GH members of the GH5 family, or can be invariant as in those belonging to GH10 (Labourel *et al.*, 2016). As an example of the glycan‐degrading abilities of a member of the HGM, the genome of *Bacteroides thetaiotaomicron* is predicted to encode 288 GHs that belong to 57 families (Lapebie *et al.*, 2019). GHs hydrolyse glycosidic bonds through an acid/base‐assisted mechanism deploying either double or single displacement catalysis, leading to the retention or inversion of the anomeric configuration, respectively. In nearly all of the 166 GH families described in the CAZY database, the catalytic apparatus involves two carboxylate residues being either aspartic or glutamic acid (Terrapon *et al.*, 2018).

All sequenced *Bacteroides* genomes have been shown to harbour so‐called polysaccharide utilization loci (PULs), which consist of clustered and co‐regulated genes encoding CAZymes, glycan transporters and sensors that control transcription of the cognate locus (Foley *et al.*, 2016; Glenwright, *et al.*, 2017). Generally, a cell surface‐located GH or PL initiates glycan degradation. The oligosaccharides that are in this way generated are imported into the periplasm and then further depolymerized by periplasmic GHs and/or PLs into monosaccharides, which are then internalized into the cytoplasm and used as energy and carbon sources (Ndeh, *et al.*, 2017). Transcriptomic data identified two *Ba. thetaiotaomicron* PULs activated by GA‐AG (Cartmell, *et al.*, 2018). Biochemical characterization of the proteins encoded by the two associated genetic loci generated models for how GA‐AG is degraded by the bacterium. Briefly, the backbone is depolymerized by a series of exo‐β‐1,3‐galactosidases and the liberated backbone‐associated side chains (these enzymes accommodate side chains being appended at the O6 position of the backbone galactose units) are then removed by β‐1,6‐galactosidases, α‐l‐rhamnosidases, β‐d‐glucuronidases and arabinosidases through a hierarchical exo‐mechanism. However, upon extracellular degradation of GA‐AG *Ba. thetaiotaomicron* releases particular oligosaccharides into the media, apparently because it does not possess the enzymatic machinery to internalize and/or metabolize them (Cartmell, *et al.*, 2018). The proposed AG degradation models highlight the fact that to fully understand the metabolism of these complex AG glycans, we need to explore the nature of these released oligosaccharides as they may feed other bacteria in the human gut where competition for carbon resources is very high.

*Bifidobacterium breve* UCC2003 has been reported to metabolize certain human milk oligosaccharides, as well as various other dietary glycans, such starch, raffinose, cellodextrins and galactooligosaccharides (Izumi, *et al.*, 2019; James, *et al.*, 2019a; James, *et al.*, 2019b; Ruiz‐Aceituno *et al.*, 2020). However, *B. breve* is generally not able to degrade and metabolize (complex) polysaccharides (O’Connell Motherway, *et al.*, 2018). It is therefore believed that *B. breve* scavenges oligosaccharides that are released from complex polysaccharides by the action of a primary degrader. This cross‐feeding or syntrophic behaviour is known to occur between species of the Bacteroidetes and Firmicutes phyla, although members of the *Bifidobacterium* genus, which belongs to the Actinobacteria phylum, may also engage in such cross‐feeding events (Turroni, *et al.*, 2018; Singh, 2019; Gutierrez and Garrido, 2019). With respect to AG utilization, it has been shown that only three *Bacteroides* species are able to utilize GA‐AG. In co‐culture (Degnan and MacFarlane, 1995; Macfarlane, *et al.*, 1995), these three organisms (which for this reason were designated as keystone organisms) enabled 17 other *Bacteroides* species to grow on GA‐AG through syntrophy by the utilization of oligosaccharides that are generated at the cell surface of the keystone bacteria.

In the current study, we demonstrate that growth of *Bacteroides cellulosilyticus* on LW‐AG releases rhamnose and ß‐1,3‐galactooligosaccharides with a varying degree of polymerization (DP) that can be metabolized by a selected few bifidobacteria, including *B. breve* UCC2003. The metabolic pathway that allows *B. breve* UCC2003 to perform this cross‐feeding behaviour is identified and characterized.

## Results

### Cross‐feeding between Bacteroides cellulosilyticus DSM 14838 (Baccell) and various Bifidobacterium species

**Fig. 1:**
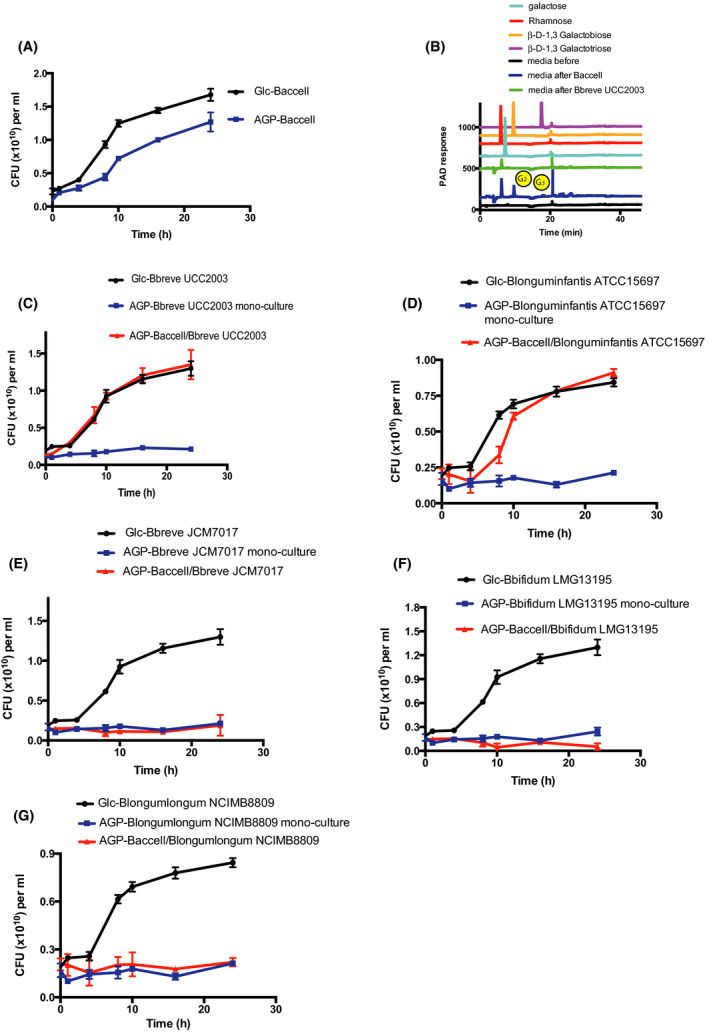
Growth of *Bacteroides cellulosyliticus* (Baccell) and several bifidobacterial species. A. Growth curves of Baccell on glucose and LW‐AG. B. HPLC of the supernatant of Baccell grown on LW‐AG where G_2_ is β‐1,3‐galactobiose and G_3_, β‐1,3‐galactotriose. C. Colony‐forming units (CFU) of *B. breve* UCC2003 for its co‐culture with Baccell on glucose and LW‐AG. D. CFU of *B. longum* subsp*. infantis* ATCC15697 for its co‐culture with Baccell on glucose and LW‐AG. E. CFU of *B. breve* JCM7017 for its co‐culture with Baccell on glucose and LW‐AG. F. CFU of *B. bifidum* LMG13195 for its co‐culture with Baccell on glucose and LW‐AG. G. CFU of *B. longum* subsp*. longum* NCIMB8809 for its co‐culture with Baccell on glucose and LW‐AG. All experiments were made in triplicate and the mean ± standard deviation is displayed.

In order to assess possible cross‐feeding behaviour between Baccell and *Bifidobacterium* strains, we initially selected the prototype strain *B. breve* UCC2003 as a candidate to determine if it could metabolize the oligosaccharides released by Baccell when grown on LW‐AG, as previously established (Cartmell, *et al.*, 2018). For this purpose, we performed a cross‐feeding experiment where *B. breve* UCC2003 was grown on its own or in conjunction with Baccell, a so‐called keystone microorganism capable of metabolizing AGs (Cartmell, *et al.*, 2018). In this experimental design, we counted the colony‐forming units (CFU) of each taxa by selection on specific growth media and antibiotics. For example, we used brain heart infusion (BHI) medium supplemented with haematin when we assessed *Bacteroides* CFU, while for bifidobacterial species, we selected on Reinforced Clostridium Media medium (see Experimental procedures section). Figure 1A shows the CFU‐based growth profile of Baccell on glucose and intact LW‐AG, confirming that this bacterium is able to metabolize the latter glycan, as a result of which a monosaccharide and certain oligosaccharides are released into the growth medium as shown in Fig. 1B. The most abundant among these released oligosaccharides were shown to be rhamnose, 1,3‐galactobiose and 1,3‐galactotriose, as identified by HPAEC‐PAD (Fig. 1B). *B. breve* UCC2003 is unable to grow on intact LW‐AG (Fig. 1C), whereas co‐cultivation experiments of Baccell and *B. breve* UCC2003 on LW‐AG as a sole carbon source showed that the latter strain is able to grow (Fig. 1C), thus clearly demonstrating cross‐feeding behaviour. *B. breve* UCC2003 is presumed to avail of some of the released carbohydrates, with the observed growth apparently due to metabolism of β‐1,3‐galactobiose and β‐1,3‐galactotriose, rather than rhamnose, which does not appear to be a growth substrate for *B. breve* UCC2003 (Fig. 1B).

In the same way as described for *B. breve* UCC2003, we performed cross‐feeding experiments between Baccell and *B. breve* JCM7017, *B. longum* subsp*. longum* NCIMB8809, *B. longum* subsp*. infantis* ATCC15697 and *B. bifidum* LMG13195. As shown in Fig. 1, only *B. longum* subsp*. infantis* ATCC15697 is able to grow in conjunction with Baccell on LW‐AG (Fig. 1C, D). In contrast, *B. breve* JCM7017 (Fig. 1E), *B. bifidum* LMG13195 (Fig. 1F) and *B. longum* subsp*. longum* NCIMB8809 (Fig. 1G) lack this syntrophic ability. As we will show below, this inability is linked to the presence or absence of the *bga* cluster.

### Identification and sequence analysis of BgaA

In order to identify the gene or genes that allow certain bifidobacteria to cross‐feed on the released AG‐derived oligosaccharides (Fig. 1), we selected the genome of *B. breve* UCC2003 to look for β‐galactosidase‐encoding genes. Through nucleotide sequence analysis of putative genes, we identified *bgaA* (corresponding to locus tag Bbr_0285) on the genome of *B. breve* UCC2003 as a gene encoding a potential exo‐β‐galactosidase. BgaA is predicted to encode a 606 residue protein containing a catalytic domain from residues 18 (Aspartic acid) to isoleucine 461 resembling members of the glycoside hydrolase family 2 (GH2). The BgaA‐encoding gene is part of a three‐gene cluster in *B. breve* UCC2003 (Fig. S1A): (i) *bgaA*, encoding the aforementioned GH2 member BgaA, (ii) *bgaR*, specifying a transcriptional regulator of the LacI family (corresponding to locus tag Bbr_0283) and (iii) a gene predicted to encoded a sugar symporter (corresponding to locus tag Bbr_0284, designated here as *bgaB*). The presence or absence of (homologs) of the cluster in the tested strains for cross‐feeding was shown to correspond with their syntrophic ability/inability (see below for a full analysis of the prevalence of the *bga* gene cluster in bifidobacterial genomes).

In order to enzymatically characterize the *bgaA* gene product, we cloned *bgaA* and overexpressed and purified its gene product in *Escherichia coli* BL21 (DE3), see Experimental procedures section, obtaining a yield of approximately 20 mg l^−1^ of purified BgaA. We performed enzymatic assays to assess the catalytic action of this protein on several polysaccharides (Fig. S1B), including pectin β‐galactan, in which the backbone glycan is β‐1,4‐linked, type I AG from potato (where the galactan linkage is also β‐1,4), β‐1,3‐galactan and type II AG, which contains β‐1,3/1,6‐linkages in the galactan backbone.

**Fig. 2:**
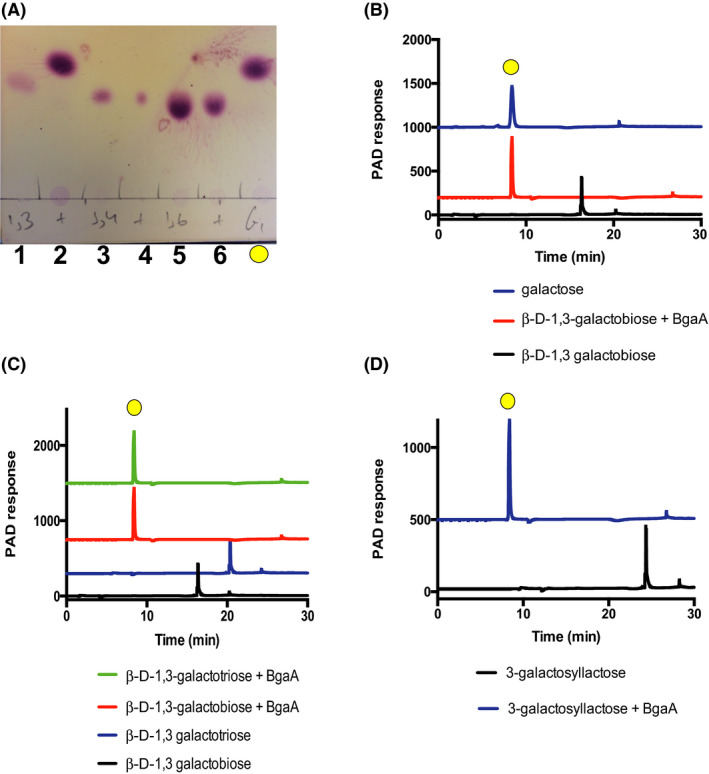
Enzymatic assays of BgaA with different disaccharides, trisaccharides and tetrasaccharides. The experimental conditions were as follows: 20 mM sodium phosphate buffer pH 7.0, [S]_0_ = 1 mM and [E]_0_ = 1 µM. A. TLC. 1 = β‐D‐1,3‐galactobiose, 2 = β‐D‐1,3‐galactobiose + BgaA, 3 = β‐D‐1,4‐galactobiose, 4 = β‐D‐1,4‐galactobiose + BgaA, 5 = β‐D‐1,6‐galactobiose 6 = β‐D‐1,6‐galactobiose + BgaA, yellow circle = galactose standard. B. HPLC of β‐D‐1,3‐galactobiose + BgaA. C. HPLC of the enzymatic assays of BgaA with β‐D‐1,3‐galactooligosaccharides. D. HPLC of the enzymatic assays of BgaA with 3‐galactosyllactose.

In addition, we performed enzyme activity reactions with particular disaccharides as potential substrates for BgaA, including β‐d‐galactopyranosyl‐4‐d‐glucopyranose (lactose), β‐d‐galactopyranosyl‐4‐*N*‐acetyl‐d‐glucosamine (LacNAc), β‐d‐galactopyranosyl‐3‐*N*‐acetyl‐d‐glucosamine (lacto‐N‐biose I), β‐d‐galactopyranosyl‐3‐d‐galactopyranose (β‐d‐1,3‐galactobiose), β‐d‐galactopyranosyl‐4‐d‐galactopyranose (β‐d‐1,4‐galactobiose), β‐d‐galactopyranosyl‐6‐d‐galactopyranose (β‐d‐1,6‐galactobiose) and β‐d‐(β‐d‐3‐O‐Galactopyranosyl)‐galactopyranosyl‐4‐d‐glucopyranose (3‐galactosyllactose) (Table 1 and Fig. S1C). These assays revealed that of the tested substrates purified BgaA only exhibits hydrolytic activity towards β‐d‐1,3‐galactobiose, indicating that this protein possesses very specific activity with a *k*_cat_/*K*_m_ of 32 580 ± 1673 M^‐1^ min^‐1^. Figure 2A,B show the TLC and HPLC results for these enzymatic reactions.

**Table 1:** Enzyme activity of BgaA with different galactooligosaccharides.

Substrate	*k*_cat_ */K*_m_ (M^‐1^ min^‐1^)
1,3‐G_2_	32 580 ± 1673
1,3‐G_3_	28 954 ± 1002
1,3‐G_4_	Inactive
3‐galactosyllactose	3030 ± 149
1,4‐G_2_	Inactive
1,6‐G_2_	Inactive
Lactose	Inactive
LacNAc	Inactive
Lacto‐N‐biose I	Inactive

### Enzyme characterization: Mode of action on β‐1,3‐galactooligosaccharides

To elucidate the enzyme specificity and the number of subsites in the active site of BgaA, we performed enzymatic assays with several 1,3‐linked galactooligosaccharides, including β‐d‐1,3‐galactobiose, β‐d‐1,3‐galactotriose, β‐d‐1,3‐galactotetraose and the human milk oligosaccharide 3’‐galactosyllactose (Fig. 2C,D). BgaA was shown to be active on β‐1,3‐galactooligosaccharides with a DP from 2 to 3 with a similar specific activity for both oligosaccharides, whereas no activity was observed for related substrates with a DP of four or higher (Table 1). This implies that BgaA harbours three subsites within its active site (−1, +1 and + 2). Regarding the + 2 subsite, when 3’‐galactosyllactose was tested as a substrate, in which the corresponding monosaccharide and linkage are β‐1,4‐galactose attached to glucose, the observed activity was decreased by ~ 10 fold, supporting the enzyme’s specificity for β‐1,3‐galactose attached to galactose. Together, our findings clearly show that BgaA exhibits very high hydrolytic specificity towards short β‐1,3‐galactooligosaccharides.

### pH profile and metal‐dependent activity for BgaA

In order to optimize the enzymatic conditions for the assays, we performed reactions to assess the behaviour of the enzyme at varying pH values (Fig. S2A). Specific activity of BgaA as a function of pH showed a typical bell shape with maximum activity at pH 7.0. This activity drastically decreased when the pH was above 7.5 and decreased when assays were performed below pH 6.0.

In addition to the pH profile, we performed additional enzymatic assays to determine if BgaA activity is metal‐dependent. For this purpose, we first treated the enzyme with EDTA in excess to remove putative metal ions from its active site and perform assays with this EDTA‐treated enzyme (Fig. S2B). BgaA was shown to lose all activity following treatment with EDTA, indeed consistent with a metal‐dependent activity. This activity was recovered when we removed the EDTA from the enzyme and added Mg^+2^ to the reaction, confirming that BgaA is an Mg^+2^‐dependent galactosidase. Enzymatic activity of EDTA‐treated BgaA was not recovered when the assays were performed with Ca^+2^, Mn^+2^ or Zn^+2^ (Fig. S2B).

### Active site comparison with other GH2 enzymes

Based on the enzyme characterization and specificity determination, to assess structural aspects of BgaA including its active site, we made several unsuccessful attempts to crystallize the molecule to obtain its 3D‐structure. As an alternative, since BgaA exhibits 25% amino acid similarity to a β‐galactosidase from *E. coli* (LacZ, database code 1JYN), we used this 1JYN structure for the inactive mutant of LacZ in complex with lactose as a template to model the BgaA structure. The conserved residues in the active site of BgaA are shown in Fig. S3A in green and the residues for LacZ in blue. In LacZ, the nucleophile E537 has been mutated to an inactive glutamine to obtain the complex with lactose. The nucleophilic residue (E514 in BgaA and E537 in LacZ) is conserved in both proteins and in the same orientation to mount the nucleophilic attack with a 7.1 Å distance with the glycosidic bond in lactose (Fig. S3A). However, the predicted catalytic donor (D421 in BgaA) is not conserved in these two proteins being E461 in LacZ. In addition, D421 is in a different orientation to E461 in LacZ having a distance of 4.3Å.

To understand the substrate specificity of BgaA towards 1,3‐β‐galactooligosaccharides, we compared the surface structure of this protein with that of LacZ. LacZ contains a deep pocket to perfectly accommodate lactose and other galactooligosaccharides, as outlined in Fig. S3B, in which the catalytic residues are highlighted in magenta. However, this pocket is not present in BgaA showing clashes in the active site when we model lactose (Fig. S3C). This effect would explain the specific activity of BgaA for β‐1,3‐linkages rather than β‐1,4 or β‐1,6 glycosidic bonds.

### Disruption of genes in the bga cluster in B. breve UCC2003

**Fig. 3:**
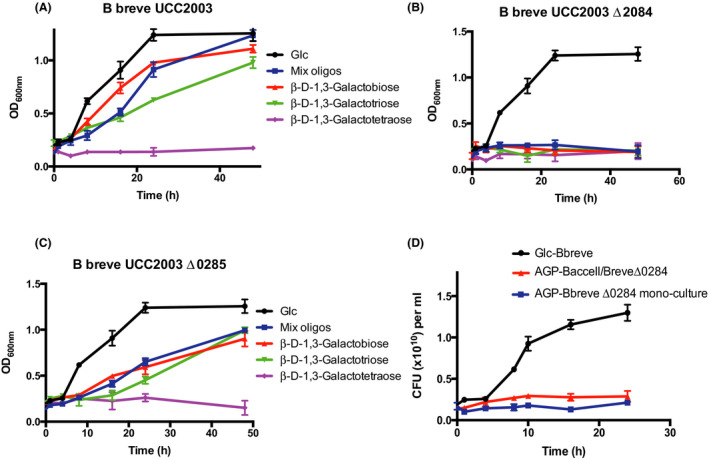
Growth curves of *B. breve* UCC2003 or its derivatives with β‐d‐1,3‐oligosaccharides. A. Wild type. B. *B. breve* BbrUCC2003Δ0284. C. *B. breve* BbrUCC2003Δ0285. D. CFU of BbrUCC2003Δ0284 for its cross‐feeding co‐cultures with Baccell on glucose and LW‐AG. All experiments were made in triplicate and the mean ± standard deviation is displayed.

In order to confirm the expected biological role and importance of Bbr_0284 (BgaB) and BgaA in the metabolism and cross‐feeding on short‐chain β‐1,3‐galactooligosaccharides, we obtained genetic knockouts for the corresponding two genes *bgaB* and *bgaA* (see Experimental procedures), and then performed growth curves employing wild‐type *B. breve* UCC2003 and the, respective, knockout mutants BbrUCC2003Δ0284 and BbrUCC2003Δ0285 (Fig. 3A–C). The wild‐type strain UCC2003 grew well in medium containing with glucose (positive control), galactobiose or galactotriose as its sole carbon source, but was unable to grow in a medium containing galactotetraose as the sole carbon source (Fig. 3A). Both mutants grew equally well on glucose as the WT UCC2003 strain, yet mutant BbrUCC2003Δ0284 (Fig. 3B) was unable to grow on galactobiose or galactotriose, while growth of mutant BbrUCC2003Δ0285 on these two galactooligosaccharides was noticeably affected in that growth rate was reduced (Fig. 3C). The latter observation is likely due to the presence of one or more additional β‐galactosidases in *B. breve* UCC2003 that are able to degrade these β‐1,3‐galactooligosaccharides, though with an apparently lower specificity. Indeed, at least eight predicted β‐galactosidases are encoded in *B. breve* UCC2003, of which some have been characterized and shown to have overlapping specificities (O’Connell Motherway, *et al.*, 2010; O’Connell Motherway, *et al.*, 2012; Ambrogi, *et al.*, 2019; James, *et al.*, 2019a; James, *et al.*, 2019b). This result indicates that the gene *bgaB* encodes a transport system that is highly specific for β‐1,3‐galactobiose and β‐1,3‐galactotriose (Fig. 3B), in the absence of which the strain is unable to internalize and consequently metabolize these saccharidic substrates.

In order to determine to what extent the observed syntrophic activities of *B. breve* UCC2003, described in Fig. 1A–C, are dependent on the *bga* cluster, we cultured *B. breve* mutant BbrUCC2003Δ0284 with Baccell together in a medium containing LW‐AG. Under these conditions, the *B. breve* mutant was unable to grow, apparently because of its inability to import the released β‐1,3‐galactooligosaccharides (Fig. 3D).

### Analysis of metabolites generated by β‐1,3‐galactooligosaccharide metabolism

Human gut microbiota interacts with the host releasing specific short‐chain fatty acids (SCFAs), mainly acetate, butyrate and propionate (Wang, *et al.*, 2019). In this sense, the determination of released SCFAs will provide insights into any benefit that the host may derive from such microbial syntrophy. LC/MS of the supernatants generated during the co‐cultivation of Baccell and *B. breve* UCC2003 is shown in Table 2. This metabolite profile shows that Baccell mainly produces succinate and acetate. This fermentation end‐product profile has previously been observed for this species when it metabolizes other carbohydrates like inulin or fructan (Falony, *et al.*, 2009). In the case of LW‐AG, the monoculture of Baccell results in acetate production due to fermentation of carbohydrates that originate from LW‐AG. When we co‐cultivated Baccell and *B. breve* UCC2003, a clear increase of acetate levels was observed when compared to that for Baccell mono‐cultivation, an observation which indicates that the overall SCFA production increases due to syntrophic behaviour.

**Table 2:** Millimolar (mM) concentration of the metabolites generated in the cell‐free supernatant of MM + 1% LW‐AG, following 24 h incubation with Baccell and *B. breve* UCC2003.

	Succinate (mM)	Acetate (mM)	Lactate (mM)	Formate (mM)
No inoculum 1% LW‐AG	ND	1.2	2.1	0.9
Baccell 1% LW‐AG	60	24	ND	ND
*B. breve* UCC2003 1% LW‐AG	ND	1.4	1.9	1.1
Baccell, *B. breve* UCC2003 1% LW‐AG	68	94	6.0	6.9

Butyrate production during co‐cultivation appeared to be similar irrespective as to whether Baccell was grown on LW‐AG in monoculture or in conjunction with *B. breve* UCC2003, confirming the known inability of *B. breve* UCC2003 to produce this SCFA. Furthermore, the co‐cultivation caused production of lactate and formate as additional metabolic end products, both of which can be exclusively attributed to *B. breve* UCC2003 metabolism (Table 2) (Watson, *et al.*, 2013; McLaughlin, *et al.*, 2015). In conclusion, the observed syntrophic interaction leads to higher and more varied levels of SCFAs and the production of lactic acid.

### Prevalence of the bga cluster among bifidobacteria

**Fig. 4:**
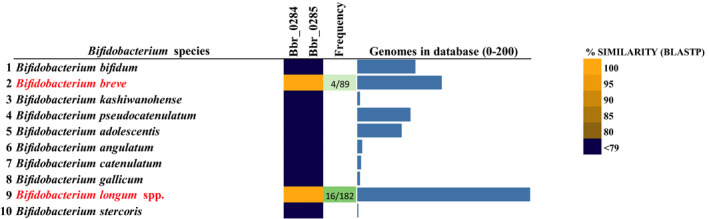
Prevalence of the *bga* cluster in *Bifidobacterium*. Heat‐map showing the BLASTP‐mediated comparative analysis of BgaA/BgaB across representatives of human‐derived bifidobacteria. Gene products from the representative strain genomes of all known publicly available *Bifidobacterium* species were scrutinized and the result of human‐derived *Bifidobacterium* species with a significant homology of 80% iterative similarity over 50% of protein length is represented in this matrix. Gene presence (yellow) and absence (blue) with a colour grading representing the degree of sequence similarity at protein level are indicated, along with the frequency of occurrence of this cluster in each species and the total number of genomes in each species.

In order to investigate the prevalence of the *bga* cluster in several *B. breve* genomes, we performed a BLASTP‐mediated search for homologues of *bgaB/bgaA* in *B. breve* and revealed that this cluster is present in just a few representatives of this species, with positive matches in only four representatives out of the 89 *B. breve* genomes accessible in the NCBI public database (Fig. 4). Interestingly, in *B. breve* strains that do not harbour the *bga* cluster, this genetic region is replaced by a carbohydrate utilization cluster of unknown specificity and predicted to encode an ABC‐type uptake system, an α‐galactosidase (glycoside hydrolase family 36) and an AraC‐type transcriptional regulator (Fig. S1A provides a comparative genomic analysis of *B. breve* strains UCC2003 and JCM 7017).

When extending this comparison to other members of the genus *Bifidobacterium*, our analysis revealed that homologues of the *bgaB/bgaA* cluster are observed in *B. longum*, with positive matches in 16 out of the 182 publicly available *B. longum* genomes. Down to the subspecies level, our analysis showed that of the 16 positive strains carrying this cluster, at least half are deposited in NCBI as members of *B. longum* subsp. *infantis* (the other half being generically referred as *B. longum*). Taken together, our analysis shows that the ability of degrading β‐1,3‐galactooligosaccharides represents an infrequently occurring feature in *Bifidobacterium*, being confined to just a few members of *B. breve* and *B. longum* subsp. *infantis* (Fig. 4).

This result is also supported by our previous observation of the distribution of the β‐galactosidase‐encoding genes from *B. breve* UCC2003 across 34 representatives of *B. breve*, *B. longum* and *B. bifidum* (Ambrogi, *et al.*, 2019). Also in this case, the presence of the BgaA β‐galactosidase Bbr_0285 was only detected in *B. breve* UCC2003 and *B. longum* subsp. *infantis* ATCC 15697 (Ambrogi, *et al.*, 2019), thus confirming our current observations, which are now extended to a much larger data set (Fig. 4). The finding that the genome of *B. longum* subsp. *infantis* ATCC 15697 contains gene homologs of the *bga* cluster is in agreement with its observed cross‐feeding ability (see above). Conversely, the other bifidobacteria tested, that is *B. breve* JCM7017, and the *B. longum* subsp. *longum* and *B. bifidum* strains do not contain the *bga* cluster and indeed were shown to be unable to cross‐feed.

## Discussion

Syntrophic interactions between species of two genera have been described for various glycans including fructan (Falony, *et al.*, 2009), wheat arabinoxylan (Rogowski, *et al.*, 2015) and AG (Degnan and Macfarlane, 1995; Macfarlane, *et al.*, 1995). For example, *Bacteroides ovatus* supports growth of *Bifidobacterium adolescentis* strain ATCC15703 because this *Bifidobacterium* strain can only utilize linear arabino‐xylo‐oligosaccharides released by *Ba. ovatus* when cultivated on complex xylan (Rogowski, *et al.*, 2015). Therefore, growth of bifidobacterial strains on highly decorated xylans, like those found in corn, depends on an extensive repertoire of enzyme families including an GH98 endo‐xylanase of *Ba. ovatus* ATCC8483.

In the current study, we describe cross‐feeding between *Ba. cellulosilyticus* (Baccell) and *B. breve* UCC2003. *B. breve* is not able to use intact AGs as a carbon source because it lacks the catalytic apparatus to degrade this large and complex polysaccharide. In contrast, Baccell can utilize this glycan as a sole carbon resource, while also releasing certain mono‐ and oligosaccharides into the media that then become available for growth of other commensal bacteria in the gut. In our study, we showed the ability of *B. breve* UCC2003 to use short‐chain β‐1,3‐galactooligosaccharides, liberated by Baccell. The *B. breve* UCC2003 *bga* gene cluster allowing this was identified and was shown to be involved in the binding, incorporation and degradation of two particular β‐1,3‐galactooligosaccharides. BgaA, which is a member of GH2, is involved in this catabolic process and is very specific to exclusively accommodate β‐1,3‐galactobiose and β‐1,3‐galactotriose in the active site. To date, only a small number of β‐galactosidases belonging to GH2 have been described to specifically utilize these oligosaccharides in *Bifidobacterium* spp. (Yi, *et al.*, 2011; Viborgh, *et al.*, 2014; Arreola, *et al.*, 2014). *Bifidobacterium* spp*.* are excellent lactose and galacto‐oligosaccharide degraders, with some species also capable of metabolizing specific human milk oligosaccharides (James, *et al.*, 2019a; James, *et al.*, 2019b). Only a small number of bifidobacterial species are known to be able to metabolize AG (Crociani, *et al.*, 1994; Fujita, *et al.*, 2019). For example, *B. longum* subsp*. longum* has been described to ferment gum arabic, a type II AG frequently added to foods as an emulsifier (Crociani, *et al.*, 1994; Cartmell, *et al.*, 2018).

The specific activity of BgaA for particular β‐1,3‐galactooligosaccharides is likely to be due to the structural properties of its active site. We showed, through the structural model for this protein, how β‐1,4 and β‐1,6‐galactooligosaccharides would sterically clash in the active site of BgaA, when compared to other, less substrate‐specific galactosidases, which contain a loop that prevents entrance of these β‐1,3‐oligosaccharides into the active site. This loop is not present in BgaA, allowing the entrance of these particular oligosaccharides, yet clashing with 1,4/1,6‐linkages, including lactose, which is not a substrate for BgaA. However, as this is only a predicted structural model, a crystal structure of this protein is required, which may then allow protein engineering of BgaA.

**Fig. 5:**
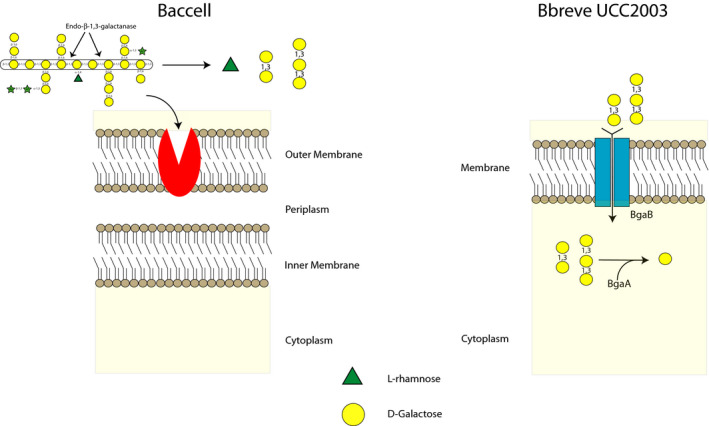
Schematic view of cross‐feeding between Baccell and *B. breve* UCC2003 when grown on LW‐AG.

In the current study, we solve the molecular interactions between Baccell and *B. breve* UCC2003 in LW‐AG metabolism, showing that Baccell mainly released rhamnose, β‐1,3‐galactobiose and β‐1,3‐galactotriose in the media (Fig. 1B). *B. breve* UCC2003 internalizes these galactooligosaccharides through BgaB into the cytoplasm, though it appears unable to incorporate and/or metabolize the released rhamnose. Following internalization of the two β‐1,3‐galactooligosaccharides, they are hydrolysed by BgaA into galactose (Fig. 5). Rhamnose may be used by other gut commensals, for example *E. coli* or perhaps other *Bacteroides species*, presenting additional syntrophic interactions (*E. coli* producing 1,2‐propanediol, which in turn can be used by for example *Eubacterium hallii* to be converted in propionic acid) (Belenguer, *et al.*, 2006; Engels, *et al.*, 2016; Matsubara, *et al.*, 2016). Therefore, the ecological complexities of LW‐AG metabolism in the gut are likely to be more elaborate than what we have demonstrated here (Fig. 5).

Remarkably, the *bga* cluster is not conserved among analysed members of the *Bifidobacterium* genus (Fig. 4). The strain‐specific presence of certain carbohydrate metabolic clusters has previously been shown to impact on colonization and persistence of particular strains in the gut (Maldonado‐Gomez, *et al.*, 2016). Therefore, strains having the *bga* cluster as presented in this paper, and the associated ability to metabolize short‐chain β‐1,3‐GOS, will provide an ecological advantage if the diet of the host contains AGs and if the microbiota houses keystone *Bacteroides *spp., such as Baccell, to provide initial breakdown and release the GOS. Baccell has been shown as a keystone organism in AG metabolism, enabling other gut microbiota species to grow in this complex glycan (Cartmell, *et al.*, 2018).

In addition to the cross‐feeding experiments and the biochemical analysis, we investigated the means by which the observed Baccell and *B. breve* UCC2003 interactions could benefit the host through SCFA production. Acidification of the colon has been shown to supress growth of pathogens, while SCFAs, which are rapidly absorbed into by the colonic mucosa, can supply energy to the host (McLaughlin, *et al.*, 2015; Connors, *et al.*, 2019). Cross‐feeding interactions between Baccell and *Bifidobacterium* when metabolizing LW‐AG, mainly leads in succinate and acetate production (Table 2). Acetate promotes the gastrointestinal motility, while succinate has been linked with protection during colonization by pathogens. Interestingly, *Bacteroides* was recently shown to mediate colonization resistance to *Salmonella typhimurium* via production of propionate, which relies on succinate as an intermediate (Connors, *et al.*, 2019).

Finally, from a prebiotic perspective it seems that this β‐1,3‐GOS represents a very species and strain‐specific carbohydrate (though we of course do not know if other gut commensal may grow on it), but it may be a very effective and highly specific synbiotic if used in combination with a probiotic that has the ability to metabolize these GOS fractions. Further studies will be required to investigate if other *Bifidobacterium* spp. or other gut commensals are able to metabolize these β‐galactooligosaccharides and how this may impact on other gut community members and, consequently and importantly, host health.

## Experimental procedures

### Bacterial strains, plasmids and culture conditions

Bacterial strains and plasmids used in this study are listed in Table S1. Where appropriate, growth media contained erythromycin (Em; 100 μg ml^‐1^) and/or kanamycin (Kan; 50 μg ml^‐1^). *Bacteroides* strains were routinely cultured in brain heart infusion (BHI) media supplemented with 1% haematin. Bifidobacterial strains were routinely cultured in either de Man Rogosa and Sharpe medium (MRS medium; Difco, BD, Le Pont de Claix, France) supplemented with 0.05% cysteine‐HCl or reinforced clostridial medium (RCM; Oxoid Ltd., Basingstoke, UK). Bifidobacterial strains were incubated under anaerobic conditions in a modular atmosphere‐controlled system (Davidson and Hardy, Belfast, Ireland) at 37°C. *Escherichia coli* strains EC101 and TUNER (DE3) were cultured in Luria‐Bertani (LB) broth (Sambrook and Maniatis, 1989), containing Kan, and without antibiotics, respectively, at 37°C with agitation. Recombinant *E. coli* EC101 cells containing (derivatives of) pFREM‐ORI28 were selected on LB agar containing Em and Kan, and recombinant *E. coli* TUNER cells containing (derivatives of) pET28b were selected on LB agar containing Kanamycin. Mutants of *B. breve* UCC2003 generated through the genomic integration of pFREM‐ORI28 were selected on reinforced clostridial agar (RCA; Oxoid Ltd.), containing Em (see below for further details on the mutation strategy). For protein overexpression, recombinant *E. coli* TUNER strains were cultured in NZY auto‐inducible LB broth (NZYTech, Lisbon, Portugal).

### Reagents

All disaccharides tested in this study were purchased from Dextra UK (Reading, UK) or Carbosynth (Reading, UK) (Fig. S1C). l‐Rhamnose, l‐arabinose, d‐Galactose, d‐glucose and Larch Wood Arabinogalactan (LW‐AGP) were purchased from Sigma (Haverhill, UK). Luria‐Bertani (LB) growth medium was purchased from Formedium (Norfolk, UK), reinforced clostridial agar from Oxoid Ltd. and brain heart infusion from Sigma. All reagents were of analytical grade.

### Cloning and expression of recombinant proteins

The gene associated with locus tag Bbr_0285 (accession number ABE94991, and designated here as *bgaA*) was amplified from *B. breve* UCC2003 using its genomic DNA as template and cloned into pET28a (Novagen, Watford, UK) using NheI and HindIII restriction sites for production and purification of its encoded product facilitated by the incorporation with an N‐terminal His_6_ tag (Table S1). For this, *E. coli* TOP10 cells (ThermoFisher Scientific) were used. The recombinant construct was sequenced (Eurofins Genomics, Ebersberg, Germany) to verify its genetic integrity and then used to transform *E. coli* BL21 (DE3) expression cells (ThermoFisher Scientific). Cells were cultured in Luria‐Bertani (LB) medium containing with 10 µg ml^−1^ kanamycin antibiotic to mid‐log phase (A_600nm_ of ~ 0.6). Protein expression was induced by adding 0.1 mM isopropyl β‐d‐1‐thiogalactopyranoside (IPTG) to cells followed by growth overnight at 16°C. The next day, cells were harvested by centrifugation (4000 *g*) and re‐suspended in Talon buffer (20 mM Tris/HCl pH 8.0 plus 100 mM NaCl). Re‐suspended cells were disrupted and centrifuged (16 000 *g*) for 20 min at 4°C, after which BgaA was purified from the resulting cell‐free extract by immobilized metal affinity chromatography (IMAC) using Talon^TM^, a cobalt‐based matrix. In the process, the cell‐free extract (CFE) was loaded on a column containing the Talon resin and then washed with Talon buffer. Another wash was performed with Talon buffer containing 10 mM imidazole followed by recombinant protein elution with 100 mM imidazole. Purified proteins were then exchanged into a buffer of choice by standard dialysis.

### Enzyme assays

All enzyme assays, unless otherwise stated, were carried out in 20 mM sodium phosphate buffer, pH 7.0, containing 150 mM NaCl and performed in triplicate. Assays were carried out at 37°C employing 1 µM BgaA in the presence of 150 µM substrate. Aliquots were taken over a 16 h time course, and samples and products were assessed by TLC and high pressure anion exchange chromatography (HPAEC) with pulsed amperometric detection (PAD). Sugars (mono and short oligosaccharides) were separated on a Carbopac PA1 guard and analytical column in an isocratic program of 100 mM sodium hydroxide during 40 min and then with a 100% linear gradient of 500 mM sodium acetate over 60 min. Sugars were detected using the carbohydrate standard quad waveform for electrochemical detection at a gold working electrode with an Ag/AgCl pH reference electrode. Kinetic parameters were determined using the d‐galactose detection kit from Megazyme, measuring the release of galactose by absorbance of 340 nm. To determine kinetic parameters, 100 nM of the appropriate enzyme was assayed against varying concentrations of oligosaccharides between 0.1 and 1 mM. d‐galactose release was measured, and the values were plotted using linear regression giving *k*_cat_/*K*_m_ as the slope of the line.

### Nucleotide sequence analysis

Sequence data were obtained from the Artemis‐mediated (Rutherford, *et al.*, 2000) genome annotations of *B. breve* UCC2003 (O’Connell Motherway, *et al.*, 2011). Database searches were performed using non‐redundant sequences accessible at the National Center for Biotechnology Information (NCBI) (http://www.ncbi.nlm.nih.gov) using the basic local alignment search tool (BLAST) (Altschul, *et al.*, 1990; Altschul, *et al.*, 1997). Sequences were verified and analysed using the SeqMan and SeqBuilder programs of the DNAStar software package (version 10.1.2; DNAStar, Madison, WI, USA). Gene product (protein) localization and signal peptide predictions were made using the TMHMM, v. 2.0 and SignalP, v. 4.1 (Petersen, *et al.*, 2011) servers, respectively, available at http://www.cbs.dtu.dk/.

### DNA Manipulations

Chromosomal DNA was isolated from *B. breve* UCC2003 as previously described (O’Riordan and Fitzferald, 1999). Plasmid DNA was isolated from *E. coli* using the Roche High Pure Plasmid Isolation kit (Roche Diagnostics, Basel, Switzerland). An initial lysis step was performed using 30 mg ml^−1^ of lysozyme for 30 min at 37°C prior to plasmid isolation from *B. breve*. DNA manipulations were essentially performed as described previously (Sambrook and Maniatis, 1989). All restriction enzymes and T4 DNA ligase were used according to the supplier’s instructions (Roche Diagnostics). Synthetic single‐stranded oligonucleotide primers used in this study (Table S1) were synthesized by Eurofins. Standard PCRs were performed using Phusion Green Hot Start II High‐Fidelity PCR Master Mix (Thermo Scientific, Waltham, MA, USA) in a Life Technologies Proflex PCR System (Thermo Scientific). PCR products were visualized by staining using SYBR Safe DNA gel stain following agarose gel electrophoresis (1% agarose). *B. breve* colony PCR reactions were performed as described previously (O’Connell Motherway, *et al.*, 2009). PCR fragments were purified using the Roche high Pure PCR purification kit (Roche Diagnostics). Plasmid DNA was isolated using the Roche High Pure Plasmid Isolation kit (Roche Diagnostics). Plasmid DNA was introduced into *E. coli* by electroporation as described previously (Sambrook and Maniatis, 1989). *B. breve* UCC2003 was transformed by electroporation according to a previously published protocol (Maze, *et al.*, 2007). The correct orientation and integrity of all plasmid constructs (see also below) were verified by DNA sequencing, performed at Eurofins.

### Construction of B. breve UCC2003 insertion mutants

Internal fragments of Bbr_0284 (designated here as *bgaB*) (fragment size was 617 bp, representing codon numbers 134 through to 340 of the 482 codons of this gene), and of *bgaA* (fragment size was 579 bp, representing codon numbers 197 through to 390 of the 607 codons of this gene), were amplified by PCR using *B. breve* UCC2003 chromosomal DNA as a template and primer pairs IM284F and IM284R, or IM285F and IM285R, respectively (Table S2) (Fig. S1A). The insertion mutants were constructed using an adaptation of a previously developed method (O’Connell Motherway, *et al.*, 2009). In short, a non‐replicating plasmid was integrated into the genome by homologous recombination, being guided by a sequence identical to that of an internal portion of the targeted gene. Successful integration of the plasmid was ensured and retained using a selectable marker of antibiotic resistance. The modification to the existing protocol was the use of an alternative plasmid with a different antibiotic resistance marker. Instead of pORI19 with an added tetracycline resistance marker, a modified version of this plasmid with an erythromycin resistance marker (*Em^R^*) (Bottacini, *et al.*, 2018) was used, designated pFREM28 (E.C. Hoedt, F. Bottacini, N. Cash, R.S. Bongers, K. van Limpt, K. Ben Amor, J. Knol, J. MacSharry and D. van Sinderen, in preparation). Site‐specific recombination of potential Em‐resistant mutant isolates was confirmed by colony PCR using primer combinations EmRFw and EmRRv to verify *Em^R^* integration, and primers 284confirm1 and 284confirm2, and 285confirm1 and 285confirm2 (positioned upstream of the selected internal fragments of *bgaB* and *bgaA*, respectively) in combination with primer EmRRv to confirm integration at the correct chromosomal location (Table S2). The data for confirmation of gene deletion are shown in supporting information.

### Cross‐feeding and competition assays

Before co‐culture, *Ba. cellulosilyticus* DSM 14838 (Baccell) was grown in BHI and washed in PBS. Monocultures of bifidobacterial strains were grown on Reinforced Clostridium Media (Oxoid) and washed in PBS before being used to inoculate Minimal Media (MM) containing 1% LW‐AG (Cartmell, *et al.*, 2018). Co‐cultures were grown in inoculate Minimal Media containing 1% LW‐AG in triplicate. Samples of 0.5 ml were taken at regular intervals during growth, which were serially diluted and plated onto brain–heart infusion (Sigma‐Aldrich) with agar and porcine haematin for determination of total CFUs per millilitre of the culture (*Bacteroides*) and onto Reinforced Clostridium Media with 0.05% cysteine (for wild‐type bifidobacterial strains) and with 100 µg ml^−1^ erythromycin (for insertional mutants in *B. breve* UCC2003). Each monoculture of *Bacteroides* or *Bifidobacterium* was also plated for determination of CFUs per millilitre at intervals during the growth.

Sugars (mixed type of oligosaccharides from growth media) were separated on a Carbopac PA200 guard and analytical column in an isocratic program of 100 mM sodium hydroxide during 40 min and then with a 100% linear gradient of 500 mM sodium acetate over a 60 min period. Sugars were detected using the carbohydrate standard quad waveform for electrochemical detection at a gold working electrode with an Ag/AgCl pH reference electrode.

### Comparative bioinformatic analysis

Protein coding sequences derived from a total of 592 representative strains from 66 bifidobacterial species were downloaded from the NCBI Reference Sequence collection Refseq (ftp://ftp.ncbi.nlm.nih.gov/genomes/refseq/bacteria) and used as input for the comparative analysis. The occurrence of *bgaA* and *bgaB* homologues (or their encoded protein products) across members of the *Bifidobacterium* genus was performed using BLASTP alignments (Altschul, *et al.*, 1997) against databases containing the Open Reading Frames (ORFs) of each bifidobacterial species. The identification of proteins homologous to BgaA and BgaB across *Bifidobacterium* was determined using cut‐off values of 80% of similarity across 50% of protein length and a 0.0001 e‐value as a significance. The result of the alignments was represented in a presence/absence binary heat‐map with a colour gradient expressing the degree of sequence similarity across bifidobacterial species, ordered by origin of isolation. A *bgaAB* gene pair was defined as being present in a given bifidobacterial genome when homologues of the two genes were found co‐located within the same genomic region with an average similarity of at least 80% at their deduced protein level.

### Metabolite analysis by High‐Performance Liquid Chromatography (HPLC)

HPLC analysis was used to assess SCFA production by (cross‐feeding) Baccell and *B. breve* UCC2003. Growth medium supernatants from stationary phase (co‐)cultures were filter sterilized (0.45 µM filter, Costart Spin‐X column) and injected into an UltiMate**®** BioRS Thermo HPLC system (Thermo Fisher Scientific) with a refractive index detector system. This system was used to identify and calculate the production of acetate, lactate and butyrate as a result of carbohydrate fermentation. Concentrations were calculated based on known standards. Non‐fermented medium containing LW‐AG served as control. An Accucore™ C18 HPLC column was used and maintained at 65°C. Elution was performed for 25 min using 10 mM H_2_SO_4_ solution at a constant flow rate of 0.6 ml min^−1^.

## Conflict of interest

None declared.

## Author contributions

J.M. and D.v.S. designed and directed the project and experiments. J.M. designed and performed the cross‐feeding and biochemical experiments. K.J. designed and performed the genetic manipulation of *B. breve* strains used in this work. F.B. performed the bioinformatics analysis of the *bga* cluster on bifidobacterial strains. J.M. and D.v.S. analysed and interpreted the data. J.M., K.J., F.B. and D.v.S. drafted the manuscript and figures, provided commentary and edits to the manuscript and figures and prepared the final version of the article.

## Supporting information


**Fig. S1**. A. bga Cluster in B. breve UCC2003 and JCM7017 strains. B. TLC of the enzymatic assays of BgaA with intact polysaccharides.PG, pectin galactan, PG +, Pectin galactan plus BgaA, LA, LW‐AGP, LA+, LW‐AGP + BgaA, GA,Gum Arabic, GA+, Gum Arabic + BgaA, 1,3G, β‐D‐1,3‐galactan, 1,3G+, β‐D‐1,3‐galactan + BgaA. C. Chemical structures of disaccharides, trisaccharides, tetrasaccharides and polysaccharides used in this work.

**Fig. S2**. A. pH‐profile of BgaA with 1,3‐galactobiose. The experimental conditions were:20 mM buffer with different pH indicated in the figure, [S]0= 1 mM and [E]0= 0.1 μM. B. Metal‐dependent activity of BgaA with β‐D‐1,3‐galactobiose. The experimental conditions were: 20 mM sodium phosphate buffer pH 7.0, [S]0= 1 mM and [E]0= 0.1 μM.

**Fig. S3**. A. Active site comparison of BgaA. B. Surface of E. coli galactosidase in complex with lactose. C. Surface of BgaA in complex with lactose.

**Table S1**. Bacterial plasmids and strains used in this work.
**Table S2**. Oligonucleotide primers used in this work.
