# Supplementary material for: Biochemical analysis of cross‐feeding behaviour between two common gut commensals when cultivated on plant‐derived arabinogalactan
Source: Microb Biotechnol. 2020 May 9;13(6):1733–47. doi: 10.1111/1751-7915.13577 (PMC7533333; doi:10.1111/1751-7915.13577)
Supplement: Supplementary file 4 — Table S1. Bacterial plasmids and strains used in this work. Table S2. Oligonucleotide primers used in this work. [file MBT2-13-1733-s004.docx]

**Supplemental Table S1.** Bacterial plasmids and strains used in this work.

| Strain or plasmid | Relevant Features  (antibiotic resistances are given in brackets) | Reference or Source |
| --- | --- | --- |
| Strains |  |  |
| *Escherichia coli* strains |  |  |
| *E. coli* EC101 | Cloning host, repA^+^ (Kan^r^) | ^O’Connell Motherway,^ *^et al.,^* ^2010^ |
| *E. coli* TUNER (DE3) | Cloning host | ^Novagen^ |
| *E. coli* TUNER (DE3)-pET28b-0285 | TUNER (DE3) containing pET28b-0285 (Kan^r^) | ^This study^ |
| *Bifidobacterium* sp. Strains |  |  |
| *B. breve* UCC2003 | Isolate from nursling stool | ^O’Connell Motherway,^ *^et al.,^* ^2010^ |
| *B. longum subsp. longum* NCIMB8809 | Isolate from human feaces | ^O’Connell Motherway,^ *^et al.,^* ^2010^ |
| *B. longum subsp. infantis* ATCC15697 | Isolate from human feaces | ^O’Connell Motherway,^ *^et al.,^* ^2010^ |
| *B. breve* JCM7017 | Isolate from nursling stool | ^O’Connell Motherway,^ *^et al.,^* ^2010^ |
| *B. bifidum* LMG13195 | Isolate from human intestine | ^O’Connell Motherway,^ *^et al.,^* ^2010^ |
| BbrUCC2003Δ0284 | pFREM-ORI28_0284 insertion mutant of UCC2003 (Em^r^) | This study |
| BbrUCC2003Δ0285 | pFREM-ORI28_0285 insertion mutant of UCC2003 (Em^r^) | This study |
| *Bacteroides* sp. Strains |  |  |
| *Bacteroides cellulosilyticus DSM14838* | Isolate from human intestine | ^Cartmel,^ *^et al.,^* ^2018^ |
| Plasmids |  |  |
| pAM5 | pBC1-puC19-(Tet^r^) | ^James,^ *^et al.,^* ^2019a^ |
| pFREM-ORI28 | Em^r^, repA^-^, ori^+^, *bbrIIIM* cloning vector (Em^r^) | [^Hoedt^](#_heading=h.35nkun2) *^et al.,^* ^submitted^ |
| pFREM-ORI28-284 | Internal 617 bp fragment of *Bbr_0284* cloned in pFREM-ORI28 (Em^r^) | ^This study^ |
| pFREM-ORI28-285 | Internal 579 bp fragment of *Bbr_285* cloned in pFREM-ORI28 (Em^r^) | ^This study^ |
| pET28b | Em^r^, IPTG-inducible expressional vector plasmid (Em^r^) | ^Novagen^ |
| pET28b-0285 | Em^r^, pET28b derivative expressing Bbr_0285 (Em^r^) | ^This study^ |

Em^r^ and Kan^r^ resistance to erythromycin and kanamycin, respectively.

UCC, University College Cork Culture Collection.

**Supplemental Table S2.** Oligonucleotide primers used in this work.

| Purpose | Primer | Sequence (5’-3’) |
| --- | --- | --- |
| Cloning of BgaA | Bbr0285f | gccatt**gctagc**atggagcgaatccaatacccc |
|  | Bbr0285r | gccgcg**aagctt**tcacacctgcacgtagccgta |
|  |  |  |
| Cloning of internal 617 bp fragment of BgaB in pORI19 | IM284F  IM284R | tgcatc**gcatgc**gctacgccttctccgacatc  tgcatc**aagctt**gccgatgaacacgaaggtc |
| Cloning of internal 579 bp fragment of BgaA in pORI19 | IM285F  IM285R | tgcatc**aagctt**gacttcacgatgaccccgc  tgcatc**gatatc**cacattggcgatgagctcc |
| Amplification of *EmR* | EmRFw  EmRRv | gtctgcatacggacacgg  ccgttgaggaggtcgttg |
| Confirmation of site specific homologous recombination | 284confirm1  284confirm2  285confirm1  285confirm2 | gacatcggcaacggcagc  cagctacggcatcatcgg  ggagcgaatccaataccccg  gaccgcaccatcgaggtg |

Restriction sites incorporated into oligonucleotide primer sequences are indicated in bold.
